# Metastatic Patterns of Mediastinal Lymph Nodes in Small-Size Non-small Cell Lung Cancer (T1b)

**DOI:** 10.3389/fsurg.2020.580203

**Published:** 2020-09-22

**Authors:** Yijun Wu, Chang Han, Liang Gong, Zhile Wang, Jianghao Liu, Xinyu Liu, Xinyi Chen, Yuming Chong, Naixin Liang, Shanqing Li

**Affiliations:** ^1^Department of Thoracic Surgery, Peking Union Medical College Hospital, Chinese Academy of Medical Sciences & Peking Union Medical College, Beijing, China; ^2^Peking Union Medical College, Eight-year MD program, Chinese Academy of Medical Sciences, Beijing, China; ^3^Department of Radiology, Peking Union Medical College Hospital, Chinese Academy of Medical Sciences & Peking Union Medical College, Beijing, China

**Keywords:** lymph node metastasis, non-small cell lung cancer, skip metastasis, selective lymph node dissection, metastatic pattern

## Abstract

**Background:** Lymph node metastasis (LNM) status is critical to the treatment. Fewer studies has focused on LNM in patients with small-size non-small cell lung cancer (NSCLC). This study aims to investigate clinicopathological characteristics associated with skip N2 (SN2) and non-skip N2 (NSN2) metastasis, and their metastatic patterns in NSCLC with tumor size of 1–2 cm.

**Methods:** We reviewed the records of NSCLC patients with tumor size of 1–2 cm who underwent lobectomy with systematic lymph node dissection (LND) between January 2013 and June 2019. Clinical, radiographical, and pathological characteristics were compared among N1, SN2, and NSN2 groups. Metastatic patterns of mediastinal lymph node were analyzed based on final pathology.

**Results:** A total of 63 NSCLC patients with tumor size of 1–2 cm were staged as pN2, including 25 (39.7%) SN2 and 38 (60.3%) NSN2. The incidence rates of SN2 and NSN2 were 2.8% (25/884) and 4.3% (38/884), respectively. For all clinicopathological characteristics, no significant difference was observed among the groups of N1, SN2, and NSN2. For the tumor located in each lobe, specific nodal drainage stations were identified: 2R/4R for right upper lobe; 2R/4R and subcarinal node (#7) for right middle lobe and right lower lobe; 4L and subaortic node (#5) for left upper lobe; #7 for left lower lobe. However, there were still a few patients (10.9%, 5/46) had the involvement of lower zone for tumors of upper lobe and the involvement of upper zone for lower lobe.

**Conclusions:** SN2 occurs frequently in patients with small-size NSCLC. Whether lobe-specific selective LND is suitable for all small-size patients deserves more studies to confirm. Surgeons should be more careful when performing selective LND for tumors located in the lower and upper lobes.

## Introduction

An increasing incidence of small-size non-small cell lung cancer (NSCLC) has been identified in recent years (1). Lobectomy with systematic lymph node dissection remains the standard treatment for NSCLC ≤ 2 cm (2), but sublobar resection (wedge resection and segmentectomy) and non-surgical treatment have attracted growing attention. Although some studies reported no significant survival between standard lobectomy and sublobar resection (3, 4), the incidence rate of occult lymph node metastasis (LNM) was high in patients with T1a-b NSCLC (5, 6).

The new strategy of selective lymph node dissection (LND) has been considered for early-stage NSCLC. The American College of Surgery Oncology Group Z0030 study reported that no significant survival was observed between patients with early-stage NSCLC that received systematic LND and lymph node sampling. Furthermore, the sublevel dissection of lymph nodes could lead to the lower perioperative complications and morbidities in patients receiving selective LND, especially in elderly patients (7, 8). However, little is known about the extent of selective LND, which has raised more interests for early-stage NSCLC (9, 10). Therefore, it is important to elucidate the metastatic patterns of mediastinal lymph nodes in patients with NSCLC ≤ 2 cm. Previous studies reported the characteristics of mediastinal LNM in patients staged as pN2, but few of them focused on LNM in NSCLC ≤ 2 cm, and the metastatic patterns of NSCLC ≤ 2 cm remain unclear.

Pathologic N2 stage can be divided into skip N2 (SN2) and non-skip N2 (NSN2). SN2 is defined as positive mediastinal nodal involvement without positive N1 nodes (pN1), while NSN2 with positive N1 nodes. According to previous studies, the incidence rate of SN2 ranged from 17 to 53% (Table 1) (11–26). It has been reported that there was difference of prognosis and mechanisms between patients staged as SN2 and NSN2 (24, 26). Whether SN2 should be evaluated alone remains controversial. This study focuses on mediastinal LNM among patients with NSCLC ≤ 2 cm to investigate the clinicopathological characteristics and metastatic patterns of mediastinal lymph nodes.

**Table 1:** The studies of mediastinal lymph node metastasis involving skip N2 and non-skip N2.

**Name**	**Year**	**pN2 (*n*)**	**Tumor size or T stage**	**SN2**			**NSN2**			***p*-value**
				***n***	**Incidence rate**	**5-year survival** **[*n* (%)]**	***n***	**Incidence rate**	**5-year survival** **[*n* (%)]**	
Tateishi et al. (11)	1994	186	T1-4	62	33%	61 (24%)	124	67%	122 (21%)	–
Tsubota et al. (12)	1996	175	1.2–8.0 cm	29	17%	–	146	83%	–	–
Yoshino et al. (13)	1996	110	T1-3	33	30%	33 (35%)	77	70%	77 (13%)	0.054
Fukuse et al. (14)	2000	87	T1-3	30	34%	30 (51%)	57	66%	57 (33%)	0.120
Gawrychowski et al. (15)	2003	64	>3 cm	23	36%	23 (26%)	41	64%	41 (0%)	0.002
Prenzel et al. (16)	2003	45	T1-4	17	38%	17 (41%)	28	62%	28 (14%)	0.019
Tanaka et al. (17)	2004	127	T1-4	60	47%	60 (30%)	67	53%	67 (31%)	0.950
Misthos et al. (18)	2004	151	T1-3	44	29%	–	107	71%	–	–
Riquet et al. (19)	2005	731	Any size	209	29%	204 (34%)	522	71%	490 (19%)	<0.001
Benoit et al. (20)	2006	142	T1-4	42	30%	42 (37%)	100	70%	100 (48%)	0.490
Ohta et al. (21)	2006	94	T1-4	50	53%	50 (33%)	44	47%	44 (20%)	0.019
Sonobe et al. (22)	2013	496	T1-3	248	50%	248 (48%)	248	50%	248 (42%)	0.168
Gorai et al. (23)	2014	52	T1	21	40%	–	31	60%	-	-
Li et al. (24)	2015	177	Any size	45	25%	45 (61%)	132	75%	132 (32%)	0.024
Guerrera et al. (25)	2016	279	T1-4	54	19%	54 (42%)	225	81%	225 (44%)	0.840
Yazgan et al. (26)	2019	130	Any size	59	45%	59 (51%)	71	55%	71 (22%)	0.001

*SN2: skip N2; NSN2: non-skip N2*.

## Materials and Methods

### Study Population

This retrospective study reviewed 884 NSCLC patients with tumor size of 1–2 cm. TNM staging was based on the American Joint Committee on Cancer (AJCC) 8th edition TNM classification (27). All patients received lobectomy with systematic LND between January 2013 and June 2019 at Department of Thoracic Surgery, Peking Union Medical College Hospital (PUMCH). Pathological examination was according to the 2015 World Health Organization (WHO) classification (28). Patients were excluded for multiple lung nodules or receiving radiotherapy or neoadjuvant chemotherapy before surgery. This study has been approved by the Ethics Committee of PUMCH and informed written consents of all patients have been obtained.

### Clinical, Radiographical, and Histological Characteristics

Medical records of clinical information were as follows: sex, age, smoking status, and serum tumor biomarker level (carcinoembryonic antigen, CEA). Radiographical characteristic including maximal tumor size, tumor imaging density, specific signs (spiculation, vessel convergence, lobulation, pleural indentation, and calcification). The maximal tumor size was measured at the lung window level. Based on imaging density, tumors were divided into 4 groups: pGGO, mixed GGO (mGGO; solid percentage < 50%), mGGO (solid percentage > 50%) and solid nodule. The solid percentage in mGGO was defined as the ratio of the maximal tumor diameter at the mediastinal window level to that at the lung window level. CT images were reviewed by one radiologist and two thoracic surgeons independently. Consensus reading was performed by them together when disagreement occurred. All patients underwent computed tomography (CT) or positron emission tomography/computed tomography (PET/CT) within 60 days before surgery. Patients who were highly suspected lung cancer nodule on CT were recommended to undergo PET/CT examination. None of patients underwent invasion examination such as endobronchial ultrasonography and mediastinoscopy before surgery.

### Analysis of Lymph Node Metastasis

Based on IASLC lymph node map (29), the intrathoracic lymph nodes were grouped into five zones, which were supraclavicular zone (1R, 1L), upper zone (2R, 2L, 3A, 3P, 4R, 4L), aortopulmonary (AP) zone (#5, #6), lower zone (#7, #8, #9), and N1 zones (#10, #11, #12, #13, #14). The extent of systematic LND included N1 nodes (#10, #11, #12, #13, #14) and mediastinal zones (2R, 4R, 3A, 3P, #7, #8, and #9 for right lung tumor and 4L, #5, #6, #7, #8, and #9 for left lung tumor, if possible).

### Statistical Analysis

Statistical analysis was performed by using IBM SPSS 25.0 (SPSS Inc; Chicago, IL, USA). Two-category comparison was performed by Pearson's Chi square test or Fisher's exact test and quantitative data was compare using Mann-Whitney *U*-test. The non-parametric data was analyzed using Kruskal-Wallis test. Statistical significance was considered when *p* < 0.05.

## Results

### Patient Characteristics

Characteristics of 108 NSCLC patients with histologically positive lymph nodes that met our inclusion criteria were summarized in Table 2, including 48 males and 60 females, with a median age of 59 (IQR: 54–66) years. The incidence rates of N1, SN2, and NSN2 in our center were 5.1% (45/884), 2.8% (25/884), and 4.3% (38/884), respectively. Smokers were found in 27.8% (30/108) of all patients. The median tumor size on CT was 1.7 cm (IQR: 1.5–2.0). There were 60 patients that underwent PET scan, with a median tumor SUV_max_ of 5.85 (IQR: 3.50–9.20). Serum CEA level tests were performed before surgery, with a median value of 3.19 ng/ml (IQR: 2.04–6.40). The pathologic outcomes identified 93 adenocarcinomas (ADC), 11 squamous cell carcinomas (SCC), and 4 other NSCLCs. Among pN2 patients, 25 patients (39.7%, 25/63) were proved to be SN2 metastasis.

**Table 2:** The characteristics of patients with non-small cell lung cancer (≤ 2 cm).

**Characteristics**	**Patients (*n* = 108)**
**Age, years**	
Median (IQR)	59 (54–66)
**Sex**	
Male/female	48/60
**Smoking status**	
Yes/no	30/78
**Tumor location**	
Right (RUL/RML/RLL)	60 (27/14/19)
Left (LUL/LLL)	48 (31/17)
**Tumor size, cm**	
Median (IQR)	1.7 (1.5–2.0)
**SUV**_**max**_ **(*****n*** **=** **60)**	
Median (IQR)	5.85 (3.50–9.20)
**CEA, ng/ml**	
Median (IQR)	3.19 (2.04–6.40)
**Histology**	
ADC/SCC/Others	93/11/4
**Pathologic N status**	
N1	45
SN2	25
NSN2	38

*RUL, right upper lobe; RML, right middle lobe; RLL, right lower lobe; LUL, left upper lobe; LLL, left lower lobe; SUVmax, maximal standardized uptake value; CEA, carcinoembryonic antigen; ADC, adenocarcinoma; SCC, squamous cell carcinoma; SN2, skip N2; NSN2, non-skip N2*.

### Comparison of Clinicopathological Characteristics Among N1, SN2, and NSN2

To find the features that associated with SN2 and NSN2 metastasis, univariate analysis was performed among patients staged as N1, SN2, and NSN2 (Table 3). For all clinicopathological characteristics, no significant difference among these three groups was observed in our study.

**Table 3:** Comparison of clinicopathological characteristics among N1, SN2 and NSN2.

	**Lymph node status**			***p*-value**
	**N1**	**SN2**	**NSN2**	
Total	45	25	38	
**Age, years**				
Median (IQR)	57 (54–65)	64 (57–68)	59 (51–62)	0.245
**Gender**				
Male/female	18/27	11/14	19/19	0.658
**Smoking status**				
Yes/no	13/32	6/19	11/27	0.891
**Tumor side**				
Right/left	20/25	16/9	24/14	0.145
**Tumor location**				
RUL/RML/RLL	9/7/4	9/3/4	9/4/11	0.219
LUL/LLL	17/8	4/5	10/4	
**Tumor size, cm**				
Median (IQR)	1.7 (1.5–2.0)	1.8 (1.6–2.0)	1.8 (1.5–2.0)	0.698
**Tumor consistency**				
solid < 50%/≥ 50%	23/22	12/13	14/24	0.132
**Spiculation**				
Yes/no	25/20	19/6	23/15	0.234
**Vessel convergence**				
Yes/no	5/40	6/19	6/32	0.365
**Lobulation**				
Yes/no	18/27	12/13	21/17	0.38
**Pleural indentation**				
Yes/no	17/28	11/14	14/24	0.833
**Tumor SUV**_****max****_				
Median (IQR)	6.00 (3.55–8.15)	6.60 (4.10–11.90)	5.60 (3.35–9.20)	0.741
**CEA, ng/ml**				
Median (IQR)	3.19 (2.20–5.43)	4.12 (2.90–7.12)	2.74 (1.86–6.63)	0.523
**Histopathology**				
Adenocarcinoma	36	20	37	0.132
Squamous	7	3	1	
Other type	2	2	0	
**Papillary component**				
Present/absent	11/34	8/17	15/23	0.339
**Micropapillary component**				
Present/absent	21/24	8/17	17/21	0.467
**Solid component**				
Present/absent	14/31	10/15	15/23	0.658
**Acinar component**				
Present/absent	34/11	17/8	31/7	0.466
**Lepidic component**				
Present/absent	3/42	4/21	5/33	0.435
**Lymphovascular invasion**				
Present/absent	4/41	4/21	11/27	0.056
**Pleural invasion**				
Present/absent	9/36	9/16	12/26	0.290

*SN2, skip N2; NSN2, non-skip N2; RUL, right upper lobe; RML, right middle lobe; RLL, right lower lobe; LUL, left upper lobe; LLL, left lower lobe; SUV_max_, maximal standardized uptake value; CEA, carcinoembryonic antigen*.

### Distribution of Mediastinal Lymph Node Metastasis Involved With SN2 and NSN2

**Figure 1:**
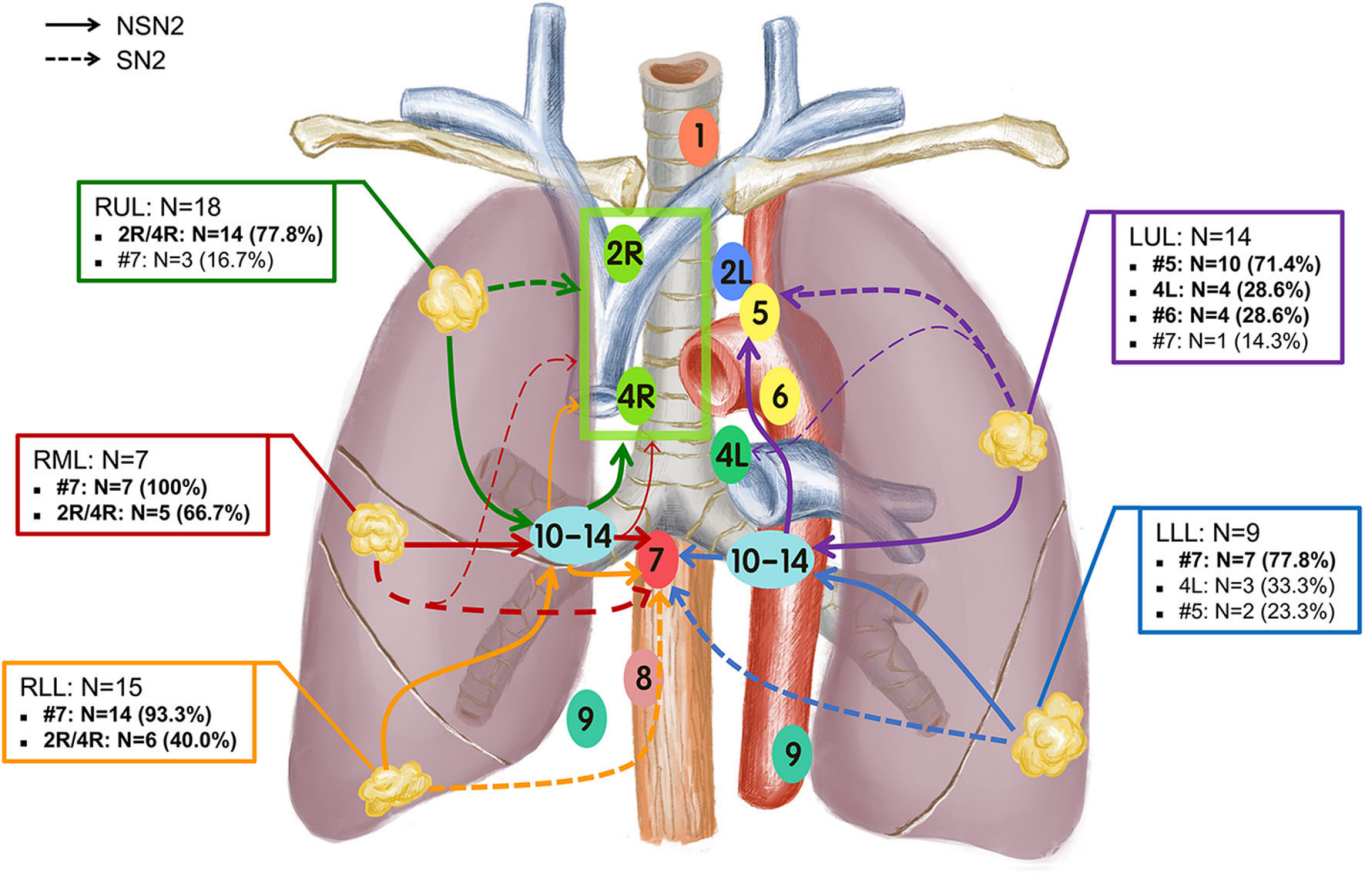
Lobe-specific nodal drainage stations in patients with small-size NSCLC (*N* = 63). The main drainage pathways of nodal metastases for each tumor location lobe were plotted using different colors.

The number of patients involved with the corresponding mediastinal stations of SN2 and NSN2 according to lobe locations is summarized in Supplementary Table 1. The average numbers of harvested lymph nodes among SN2 and NSN2 were 20.3 and 20.4, respectively, and a similar number of metastatic nodes was observed between NSN2 (*N* = 4.0) and SN2 (*N* = 3.9; Supplementary Table 1). For all patients with positive mediastinal lymph nodes, single-station metastasis was more commonly observed than multiple-station metastases. Furthermore, it seemed that mediastinal single-station metastasis occurred more in SN2 (80.0%, 20/25) than NSN2(57.9%, 22/38). For patients with a tumor located in each lobe, the mainly-involved lymph node stations were different but remained some similarity between SN2 (Supplementary Figure 1) and NSN2 (Supplementary Figure 2). In 25 patients staged as SN2 (Supplementary Figure 1), there were 9 patients involved with metastasis of upper zone for tumors of RUL (2R, 4R, or 2R/4R [could not distinguish between 2R and 4R]; 66.7%, 6/9), 3 involved with metastasis of lower zone for tumors of RML (#7; 100.0%, 3/3), 4 involved with metastasis of lower zone for tumors of RLL (#7; 100%, 4/4), 3 involved with metastasis of aortopulmonary zone for tumors of LUL (#5; 75.0%, 3/4) and 4 involved with metastasis of lower zone for tumors of LLL (#7; 80.0%, 4/5). Therefore, #7 (13/25, 52.0%) and 2R/4R (9/25, 36.0%) were the main metastatic stations of SN2. Similar results were also obtained in NSN2 patients (Supplementary Figure 2) with a tumor in RUL (2R/4R; 88.8%, 8/9), RML (#7; 75.0%, 3/4), LUL (#5; 70.0%, 7/10), and LLL (#7; 75.0%, 3/4). There were two (2/9) and one (1/9) patients with a tumor in RUL had positive #7 nodes in SN2 and NSN2, respectively. Specifically, 6 cases of NSN2 with a tumor in RLL had positive 2R/4R nodes, but none of positive 2R/4R nodes were observed among 4 cases of SN2 in RLL. For tumors of upper lobe, there were still a few of them (12.5%, 4/32) that had the involvement of lower zone (Figure 1). On the other hand, 45.8% (11/24) of patients with tumor of lower lobe had the involvement of upper zone. Specifically, all 6 cases that had a RLL tumor with positive 2R/4R were proved to have positive N1 nodes.

## Discussion

Pathological N2 (pN2) stage is crucial to the management of patients with NSCLC, which was observed in ~20–40% of all patients with NSCLC (17). In this retrospective study, 63 patients staged as N2 were identified, including 25 SN2 and 38 NSN2. Since the new surgical strategies of sublobar resection and selective lymph node dissection have been considered for early-stage NSCLC, it is crucial to reveal the metastatic patterns of mediastinal lymph nodes.

Skip N2, one special sub-stage of N2 that was not furtherly subdivided in the 8th edition TNM classification, has attracted more attention in recent years because of its better survival than non-skip N2 (15, 16, 21, 24, 26). Many studies reported very different incidence rates of SN2 among N2 patients, ranging from 17 to 53% (Table 1), but most of patients enrolled in their studies had a tumor size > 3 cm. To date, no previous study has focused on SN2 among patients with NSCLC ≤ 2 cm. In our study, 25 (2.8%, 25/884) patients were proved to be SN2, indicating a high incidence rate of SN2 (39.7%, 25/63) among pN2 patients with NSCLC ≤ 2 cm. Gorai et al. studied the clinicopathological features of SN2 among 422 patients with NSCLC ≤ 3 cm, in which 21 SN2 (40%) and 31 NSN2 (60%) patients were identified, but fewer patients had a tumor size ≤ 2 cm (23). By summarizing the studies about SN2 from 1994–2012, they found that the frequency of SN2 decreased over time and this might attribute to the technological advancement that increased the likelihood of diagnosing hilar lymph nodes. Several studies reported the higher incidence of SN2 was associated with larger size of tumors (19, 20). Based on that, the incidence of SN2 should have decreased with the increasing early-detection rate of NSCLC. However, in our study, a high frequency of SN2 in patients with NSCLC ≤ 2 cm was obtained. Considering NSN2 patients' better survival than SN2 (15, 16, 21, 24, 26), it might be postulated that SN2 was an different stage from NSN2, and these patients staged as SN2 should be evaluated alone.

Previous studies have reported several factors for SN2 such as tumor size at the lung window and mediastinal window of CT, tumor location, pathological type, and pleural invasion (11–26). However, in our study, we tried to enroll clinical, radiographical, and histological characteristics, but no significant difference was observed among these three groups for all variables (Table 3). However, in our previous study, many characteristics demonstrated significantly difference between node-positive and node-negative patients (30). Thus, this study indicated that patients with positive nodes, whether N1, SN2, or N2, shared certain consistency of manifested characteristics. Gorai et al. found that pleural invasion might be an important risk factor for N2 metastasis among cIA NSCLC (23). A hypothesis was put forward that tumors invaded toward the pleura and into the lymphatic ducts below the pleura, and metastasized to the mediastinal lymph nodes (23). Although our study did not obtain significance between mediastinal metastasis and pleural invasion because of the limited number of study population, patients with pleural invasion should be evaluated carefully, especially for some patients who have no positive lymph nodes of pathologic outcome. For them, N2 metastasis might occur.

Although the standard treatment for IA NSCLC remains lobectomy with SND (2), whether SND is necessary for small-size NSCLC is not clear. With the deepening of research on mediastinal LNM, the metastatic pattern in NSCLC has been reported based on a predictive manner of lobe-specificity, so called “lobe-specific selective lymph node dissection,” which might be sufficient for small-size NSCLC (9, 31, 32). Compared to SND, SLND is more appropriate for elderly patients or those with poor pulmonary reserve. However, lobe-specific SLND has not been widely accepted, for which one important reason is that the metastatic pattern of small-size remains unclear. In our study, based on tumor-located lobes, the metastatic stations among SN2 and NSN2 patients were analyzed, respectively (Supplementary Figures 1, 2). It could be inferred that patients staged as SN2 and NSN2 had a similar metastatic patterns of mediastinal lymph nodes. Our study indicated that there were still some tumors in lower lobes that invaded into upper zone and some in upper lobe that invaded into lower zone by skip or non-skip metastasis. Thus, lobe-specific SLND might not be always adequate for lung cancer patients, even for small-size lung cancer. For the small-size tumors in RML, the resection of 2R/4R and #7 nodes might be reasonable. In the clinic, surgeons should be more careful when performing SLND. Whether lobe-specific selective LND is suitable for all small-size patients deserves more studies to confirm.

There were also some limitations in this study. First, the number of patients enrolled in this pilot study was limited. Future studies may consider a larger or multicenter study population. Second, this is a retrospective study and data bias could not be avoided. Prospective studies are needed for further identification of metastatic patterns of small-size NSCLC in the future. Third, there were very few patients with NSCLC < 1 cm who had positive lymph nodes, and they were excluded for avoiding bias. The following studies may focus on this population in terms of nodal metastasis.

## Conclusions

In this study, 63 patients with NSCLC ≤ 2 cm were staged as pN2, including 25 SN2 (39.7%) and 38 NSN2 (60.3%). The incidence rates of SN2 and NSN2 were 2.8% (25/884) and 4.3% (38/884), respectively. The comparisons of clinicopathological characteristics among N1, SN2, and NSN2 groups indicated no significant difference. The analysis of the mediastinal LNM among pN2 patients revealed useful information about metastatic patterns of small-size NSCLC. Lobe-specific lymph node dissection may not be adequate for patients with a tumor in lower and upper lobes. Whether lobe-specific selective LND is suitable for all small-size patients deserves more studies to confirm. Surgeons should be more careful when performing selective LND for lung cancer patients, even for small-size lung cancer.

## Data Availability Statement

The raw data supporting the conclusions of this article will be made available by the authors, without undue reservation.

## Ethics Statement

The studies involving human participants were reviewed and approved by the Ethics Committee of Peking Union Medical College Hospital. The patients/participants provided their written informed consent to participate in this study.

## Author Contributions

SL, NL, YW, and JL: conceptualization. YW and CH: methodology and writing—original draft preparation. YW, JL, CH, XL, XC, and YC: formal analysis. YW, ZW, and LG: investigation. YW, SL, and NL: writing—review and editing. SL: supervision. All authors: contributed to the article and approved the submitted version.

## Conflict of Interest

The authors declare that the research was conducted in the absence of any commercial or financial relationships that could be construed as a potential conflict of interest.

## Supplementary Material

Supplementary Figure 1 Metastatic patterns of skip N2 metastasis (SN2) in patients with NSCLC ≤ 2 cm (*N* = 25). Each color of lines represents all the metastatic pathways of each tumor lobe location.

Supplementary Figure 2 Metastatic patterns of non-skip N2 metastasis (NSN2) in patients with NSCLC ≤ 2 cm (*N* = 38). Each color of lines represents all the metastatic pathways of each tumor lobe location.

Supplementary Table 1 The number of N2 stage patients with involved mediastinal lymph nodes stratified by tumor-located lung lobes.
