# Supplementary figures and images for: Metastatic Patterns of Mediastinal Lymph Nodes in Small-Size Non-small Cell Lung Cancer (T1b)
Source: Front Surg. 2020 Sep 22;7:580203. doi: 10.3389/fsurg.2020.580203 (PMC7536402; doi:10.3389/fsurg.2020.580203)

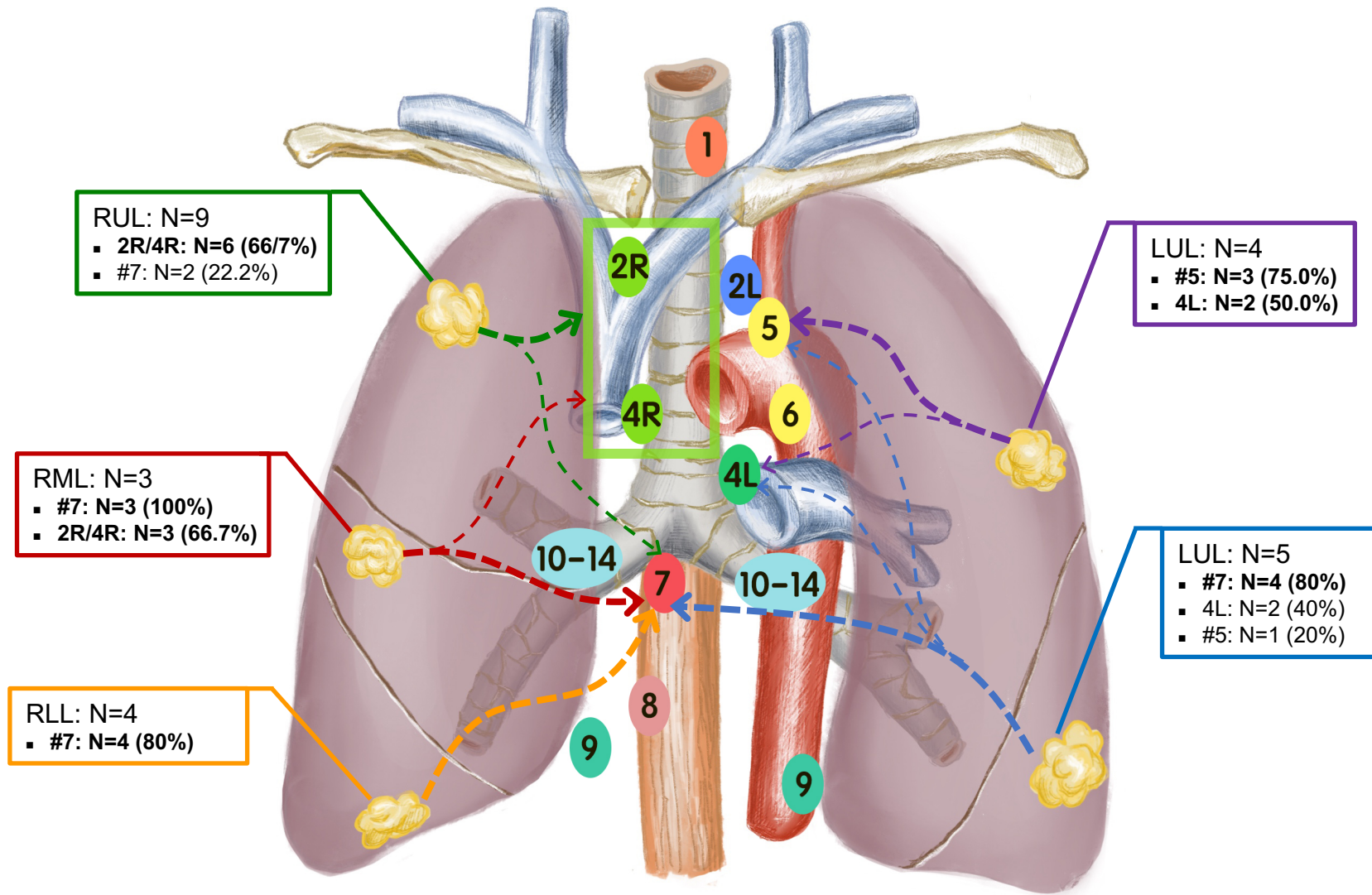

Supplement: Supplementary Figure 1 — Metastatic patterns of skip N2 metastasis (SN2) in patients with NSCLC ≤ 2 cm (N = 25). Each color of lines represents all the metastatic pathways of each tumor lobe location. [file Data_Sheet_1_v1.PDF]

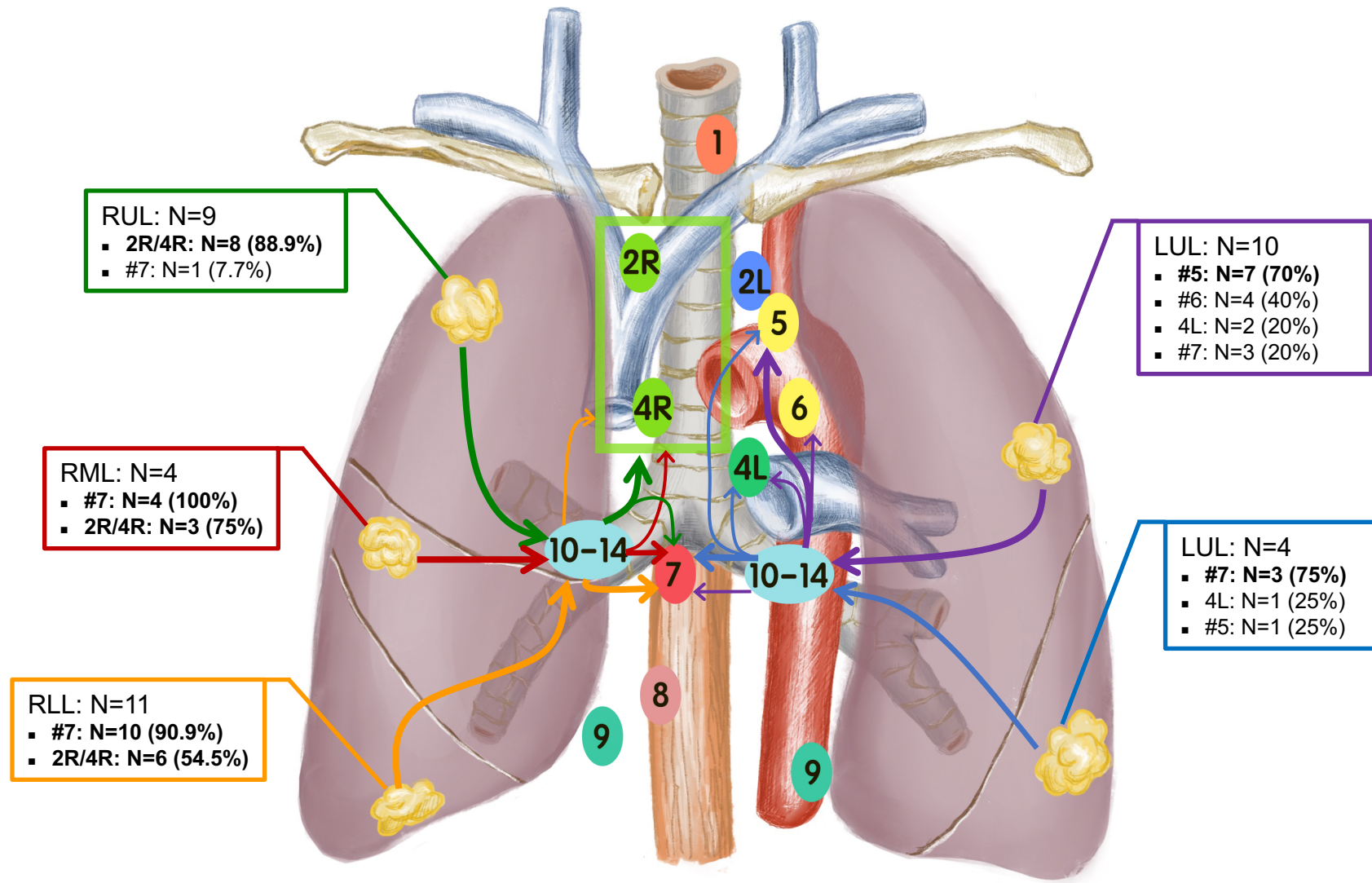

Supplement: Supplementary Figure 2 — Metastatic patterns of non-skip N2 metastasis (NSN2) in patients with NSCLC ≤ 2 cm (N = 38). Each color of lines represents all the metastatic pathways of each tumor lobe location. [file Data_Sheet_2_v1.PDF]
