# Supplementary material for: Metastatic Patterns of Mediastinal Lymph Nodes in Small-Size Non-small Cell Lung Cancer (T1b)
Source: Front Surg. 2020 Sep 22;7:580203. doi: 10.3389/fsurg.2020.580203 (PMC7536402; doi:10.3389/fsurg.2020.580203)
Supplement: Supplementary Table 1 — The number of N2 stage patients with involved mediastinal lymph nodes stratified by tumor-located lung lobes. [file Table_1.DOCX]

**Table S1. The number of N2 stage patients with involved mediastinal lymph nodes stratified by tumor-located lung lobes.**

|  | SN2 (n) | | | | | |  | NSN2 (n) | | | | | |
| --- | --- | --- | --- | --- | --- | --- | --- | --- | --- | --- | --- | --- | --- |
|  | Total  (n=25) | RUL  (n=9) | RML  (n=3) | RLL  (n=4) | LUL  (n=4) | LLL  (n=5) |  | Total  (n=38) | RUL  (n=9) | RML  (n=4) | RLL  (n=11) | LUL  (n=10) | LLL  (n=4) |
| HLN |  |  |  |  |  |  |  |  |  |  |  |  |  |
| Average | 20.3 | - | - | - | - | - |  | 20.4 | - | - | - | - | - |
| N1 nodes | 5.8 | - | - | - | - | - |  | 7.8 | - | - | - | - | - |
| Metastasis | 3.9 | - | - | - | - | - |  | 4.0 | - | - | - | - | - |
| 2R/4R* | 9 | 6 | 2 |  |  |  |  | 17 | 8 | 3 | 6 |  |  |
| 2R | 0 |  |  |  |  |  |  | 3 | 2 |  | 1 |  |  |
| 4R | 5 | 4 | 1 |  |  |  |  | 8 | 4 |  | 4 |  |  |
| 4L | 4 |  |  |  | 2 | 2 |  | 3 |  |  |  | 2 | 1 |
| #5 | 4 |  |  |  | 3 | 1 |  | 8 |  |  |  | 7 | 1 |
| #6 | 0 |  |  |  |  |  |  | 4 |  |  |  | 4 |  |
| #7 | 13 | 2 | 3 | 4 |  | 4 |  | 20 | 1 | 4 | 10 | 2 | 3 |
| Multiple-station | 5 | 1 | 2 | 0 | 1 | 1 |  | 16 | 1 | 3 | 7 | 4 | 1 |
| Single-station | 20 | 8 | 1 | 4 | 3 | 4 |  | 22 | 8 | 1 | 4 | 6 | 3 |

*including 2R, 4R or 2R/4R (nodes that cannot distinguish between 2R and 4R). HLN: number of harvested lymph nodes; SN2: skip N2; NSN2: non-skip N2; RUL: right upper lobe; RML: right middle lobe; RLL: right lower lobe; LUL: left upper lobe; LLL: left lower lobe.
